# Tentorial mobility in centipedes (Chilopoda) revisited: 3D reconstruction of the mandibulo-tentorial musculature of Geophilomorpha

**DOI:** 10.3897/zookeys.510.8840

**Published:** 2015-06-30

**Authors:** Markus Koch, Johannes Schulz, Gregory D. Edgecombe

**Affiliations:** 1 Institute of Evolutionary Biology and Ecology, University of Bonn, An der Immenburg 1, 53121 Bonn, Germany; 2 Department of Entomology, Biocentre Grindel and Zoological Museum, Martin-Luther-King-Platz 3, 20146 Hamburg, Germany; 3 Department of Earth Sciences, The Natural History Museum, Cromwell Road, London SW7 5BD, UK

**Keywords:** Evolutionary morphology, head endoskeleton, Myriapoda, skeleto-muscular system, histology

## Abstract

Mandibular mechanisms in Geophilomorpha are revised based on three-dimensional reconstructions of the mandibulo-tentorial complex and its muscular equipment in *Dicellophilus carniolensis* (Placodesmata) and *Hydroschendyla submarina* (Adesmata). Tentorial structure compares closely in the two species and homologies can be proposed for the 14/17 muscles that attach to the tentorium. Both species retain homologues of muscles that in other Pleurostigmophora are traditionally thought to cause swinging movements of the tentorium that complement the mobility of the mandibles. Although the original set of tentorial muscles is simplified in Geophilomorpha, the arrangement of the preserved homologues conforms to a system of six degrees of freedom of movement, as in non-geophilomorph Pleurostigmophora. A simplification of the mandibular muscles is confirmed for Geophilomorpha, but our results reject absence of muscles that in other Pleurostigmophora primarily support see-saw movements of the mandibles. In the construction of the tentorium, paralabial sclerites seem to be involved in neither Placodesmata nor Adesmata, and we propose their loss in Geophilomorpha as a whole. Current insights on the tentorial skeleton and its musculature permit two alternative conclusions on their transformation in Geophilomorpha: either tentorial mobility is primarily maintained in both Placodesmata and Adesmata (contrary to Manton’s arguments for immobility), or the traditional assumption of the tentorium as being mobile is a misinterpretation for Pleurostigmophora as a whole.

## Introduction

The tentorium of myriapods is a cuticular formation of the head with a distinct composition of exoskeletal bars around the mouth opening and endoskeletal processes (Koch 2003). Since Manton’s (1964) comparative studies of the head morphology in mandibulate arthropods, structural correspondences of the tentorium and its participation in movements of the mandibles are considered as the most compelling morphological evidence for the monophyly of Myriapoda (Edgecombe 2004, Shear and Edgecombe 2010). In the context of myriapod phylogeny (e.g., Edgecombe 2011), however, ambiguity remains with regard to Manton’s (1964) conclusion that the myriapod tentorium is primarily mobile, and that its “swinging” movements correlate with the presence of a mobile mandibular gnathal lobe that lacks a mandibular muscle for its abduction. This uncertainty results in particular from the presence of an apparently immobile tentorium in the basalmost offshoot among centipedes, the Scutigeromorpha, and from apparent differences among pleurostigmophoran centipedes, symphylans, and millipedes as to how tentorial movements are transferred onto the mandibles, including eventual absence of tentorial mobility in subordinate taxa within each of these groups (Verhoeff 1918, Attems 1926, 1929, Manton 1964, 1965, Desbalmes 1992).

An immobile tentorium was assumed by Manton (1965) for geophilomorph centipedes among Pleurostigmophora, based on her studies of a few representatives of the clade Adesmata. Recent comparative studies of the peristomatic structures in a broader sampling of geophilomorphs by Koch and Edgecombe (2012) challenged this assumption. They revealed anatomical evidence in support of the view that at least in the sister group to all other geophilomorphs, the Placodesmata, the mobility of the tentorium is maintained, its loss accordingly being potentially synapomorphic for adesmatan Geophilomorpha only. This preliminary conclusion still required more comprehensive anatomical studies of the mandibulo-tentorial complex, including its muscular equipment. Some of the evolutionary transformations of the tentorium in Adesmata as advocated by Koch and Edgecombe (2012) were contradicted by Bonato et al. (2014) within the scope of their analyses of geophilomorph phylogeny.

In order to contribute to a clarification of the mobility versus immobility of the tentorium in geophilomorphs, we here present anatomical 3D-reconstructions of the mandibulo-tentorial complex and its muscular system based on histological studies of two species, one representing each of the clades Placodesmata and Adesmata, respectively. Insights obtained demand a more general revision of whether the tentorium in pleurostigmophoran centipedes is mobile at all.

## Materials

**Figure 1.**
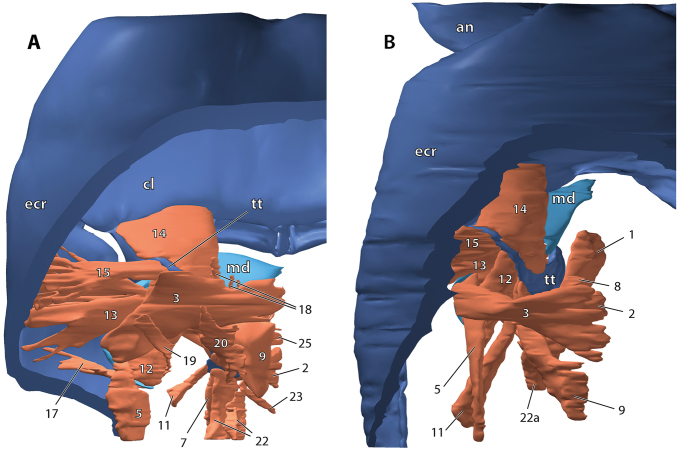
Surface model of the mandibulo-tentorial complex, dorsal view onto the left complex within the head capsule (anterior is top). **A** *Dicellophilus carniolensis* **B** *Hydroschendyla submarina*, tentorial muscles 18 and 22b removed. Numbers refer to muscles as listed in Table 1. Abbreviations: **an** antenna **cl** clypeus, **ecr** epicranium, **md** mandible, **tt** tentorium.

This study is based on histological sections of the head of *Dicellophilus carniolensis* (Koch, 1847) (Placodesmata, Mecistocephalidae) and *Hydroschendyla submarina* (Grube, 1792) (Adesmata, Schendylidae). The heads were fixed in alcoholic Bouin solution (modified according to Duboscq-Brazil) after removal of the forcipules, dehydrated in a graded ethanol series, and transferred via propylene oxide into epoxy resin (Araldite). Series of semithin transverse sections (0.5–1 µm thickness) were performed with a Jumbo-Diatome diamond knife on an Ultracut E microtome (Fa. Reichert) and stained with 1% Toluidine blue. Digital images (tiff-format) of the sections were made with an Olympus BX51dotSlide microscope and semi-automatically aligned into a digital image stack with the open source software imodalign (http://www.evolution.uni-bonn.de/mitarbeiter/bquast/software). The image stacks used for 3D-reconstructions (Fig. 1) are provided as supplementary files (Suppl. materials 1 and 2).

**Table 1.** Compilation of all mandibular and tentorial muscles, numbered in accordance to their illustration in Figs 1–5 and the 3D-reconstructions. Potential homologues in *Orya barbarica* are inferred from Manton (1965) under citation of her muscle numbering; muscles not mentioned or depicted in her work are indicated by a question mark.

Muscles (this study)	Origin	Insertion	*Dicellophilus carniolensis*	*Hydroschendyla submarina*	*Orya barbarica* (Manton 1965)
Tentorium (tt)					
9	epicranium	posterior process	+	+	? (T10)
14	clypeus	supramandibular arch	+	+	+ (T4/T5, or T2-T6)
15	epicranium	supramandibular arch	+*	+	? (T7)
18	epicranium	supramandibular arch	+	+	? (T9)
20	epicranium	posterior process	+	–	? (T8)
22	forcipular tendon	posterior process	+*	+	slm / vlm
Mandible (md)					
3	epicranium	base	+	+	26
4	base	gnathal lobe	+	+	31
5	epicranium	base	+	+	– (25, or 24+25)
6	tentorium	gnathal lobe	+	+	27
10	gnathal lobe	gnathal lobe	–	+	29
12	tentorium	base	+	+	22/23
13	epicranium	gnathal lobe	+	+	20
17	epicranium	base	+	–	19 (25 fide Manton)
19	epicranium	base	+	– (21?)	–
21	epicranium	gnathal lobe	– (19?)	+**	21
24	tentorium	base	+	+	– (30, unified with 23 fide Manton)
26	mesial inter-connection	base	–	+**	– (32)
Hypopharynx (hy)					
1	tentorium	front side along mesial lips	+	+	+
25	tentorium	back side near opening of hypoph. gland	+	+	+
Pharynx (ph)					
8	tentorium	ventral pharyngeal wall	+*	+	?
Maxilla I (mxI)					
2	tentorium	coxosternite (paramedial)	+*	+	(+)***
16	tentorium	coxosternite (lateral to 2)	+*	–	
23	tentorium	coxosternite (paramedial, posterior to 2)	+*	–	
Maxilla II (mxII)					
7	tentorium	coxosternite (medial at base of telopodite)	+*	+	(+)***
11	tentorium	coxosternite (lateral)	+*	+	

* insertion via collagenous tendon

** not shown in the 3D-reconstruction

*** depicted in Manton’s Fig. 81a but no unambiguous homologisation possible

For three-dimensional reconstructions the software Amira 5.4.5 (FEI Visualization Sciences Group) was used for segmentation. As recommended by Friedrich and Beutel (2008), the segmented volume data were imported as bmp-files into the software Bitplane Imaris 5.7 (Bitplane AG, Zürich, Switzerland) for surface rendering, smoothing and downsampling. The surfaces (inventor format) were then converted with the freeware Transform2 (Heiko Stark, Jena, Germany; http://starkrats.de) into object format for final processing with the Autodesk Maya software (Students & Educators version 2013; Alias Wavefront, Toronto/Ontario, Canada), applying the Cleanup, Average Vertices, Poly Reduce, and Smooth options to remove minor segmentation artifacts. The surface models were then exported from Maya as u3d-files (Universal 3D format) and processed with the Adobe 3D-Reviewer plug-in in Adobe Acrobat 9 Pro Extended (Adobe Systems, San Jose, California, USA) to generate an interactive pdf-file. Images and plates were edited with the Adobe Illustrator and Adobe Photoshop CS4 software.

The interactive 3D-mode can be activated by clicking on the images of Figures 2C and 3C. A tool bar then opens that allows the user to rotate, move and magnify the model, to isolate elements, and to change the background and light settings. Elements of our models comprise the head capsule, the tentorium, the mandible, and all muscles related to the tentorio-mandibular complex. Single elements can be displayed by opening the “model hierarchy” and clicking off/on the respective elements according to their label. Clicking on any element in the model hierarchy highlights the respective element in red. The “initial configuration” shows all elements in ventral view. The views of our models shown in Figures 1–3 can be displayed by choosing the respective configuration in the toolbar under “views”.

**Figure 2.**
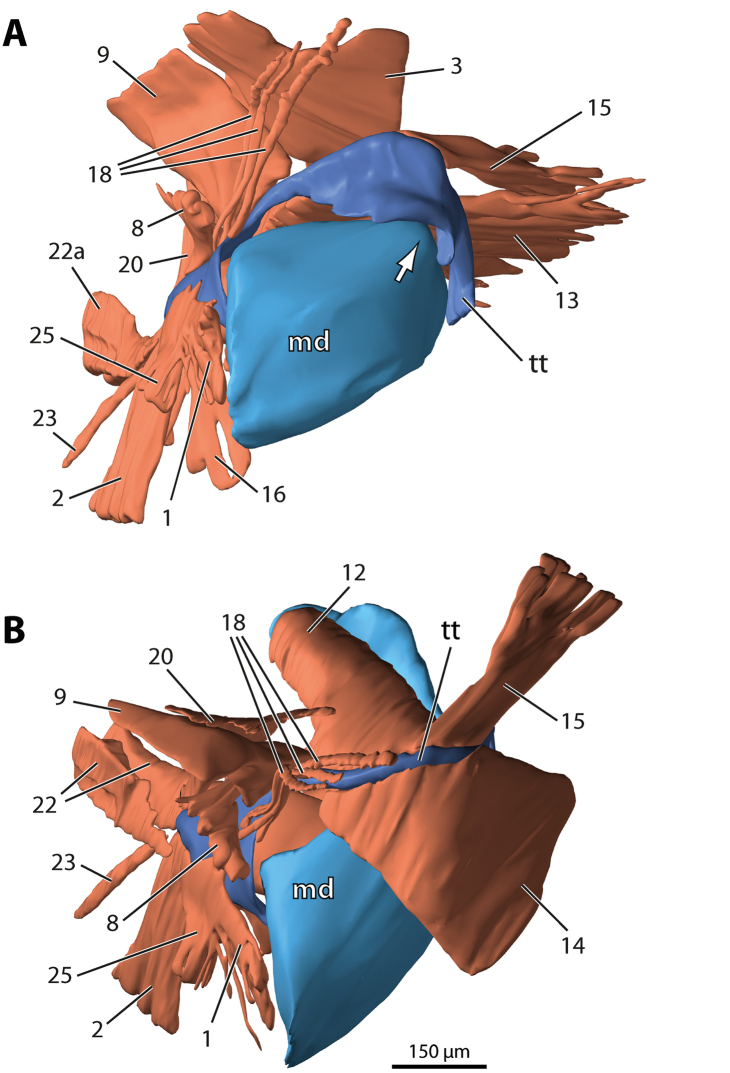
*Dicellophilus carniolensis*, surface model of the left mandibulo-tentorial complex. **A** Medio-frontal view, tentorial muscle 14 removed; arrow points to condyle of mandibular gnathal lobe **B** Oblique dorso-frontal view, extrinsic mandibular muscles removed. Numbers refer to muscles as listed in Table 1. Abbreviations: **md** mandible **tt** tentorium.

**Figure 2C.**
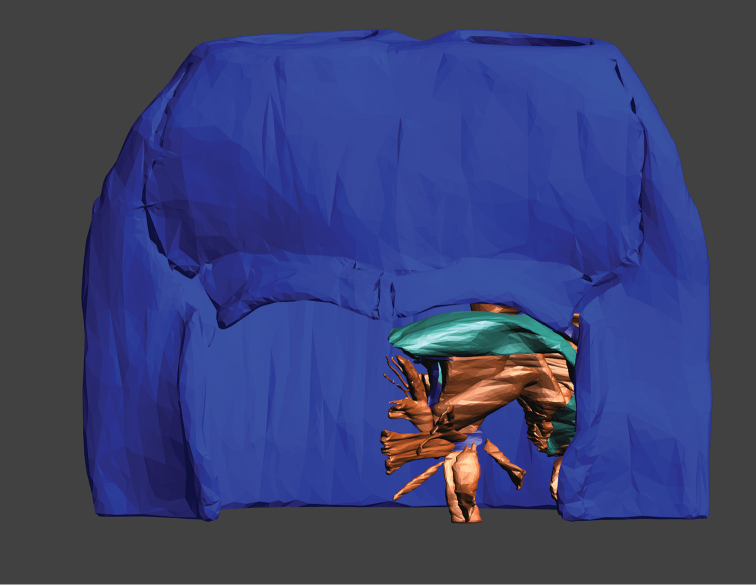
*Dicellophilus carniolensis*, surface model of the left mandibulo-tentorial complex in situ. Click on the image to activate the interactive 3D-mode. (download 3D model)

**Figure 3.**
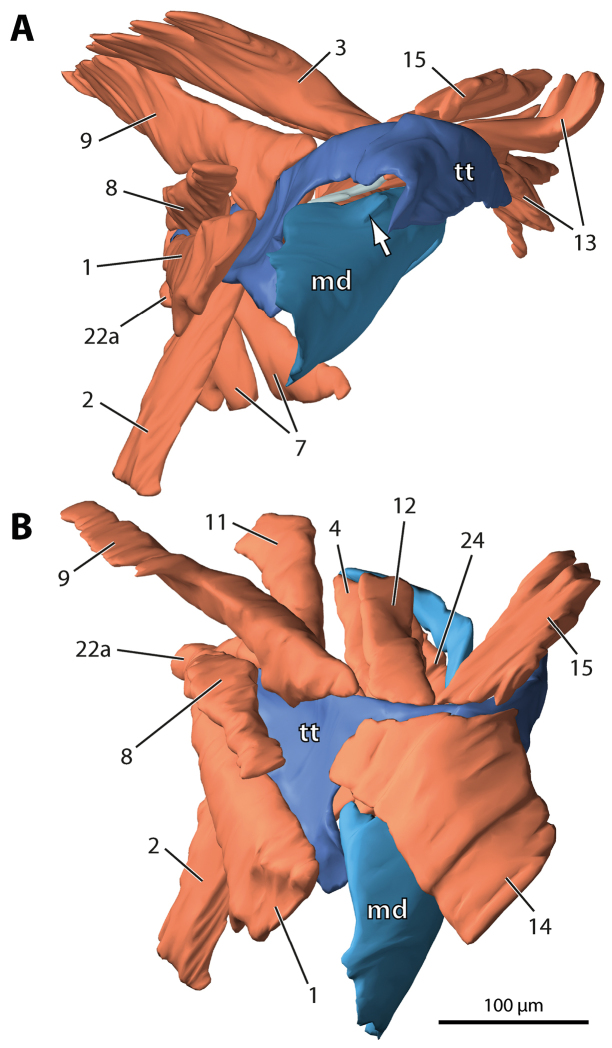
*Hydroschendyla submarina*, surface model of the left mandibulo-tentorial complex. **A** Medio-frontal view, tentorial muscles 14, 18, and 22b removed as well as hypopharyngeal muscle 25; arrow points to condyle of mandibular gnathal lobe **B** Oblique dorso-frontal view, extrinsic mandibular muscles 3, 5, and 13 removed as well as tentorial muscles 18 and 22b. Numbers refer to muscles as listed in Table 1. Abbreviations: **md** mandible **tt** tentorium.

**Figure 3C.**
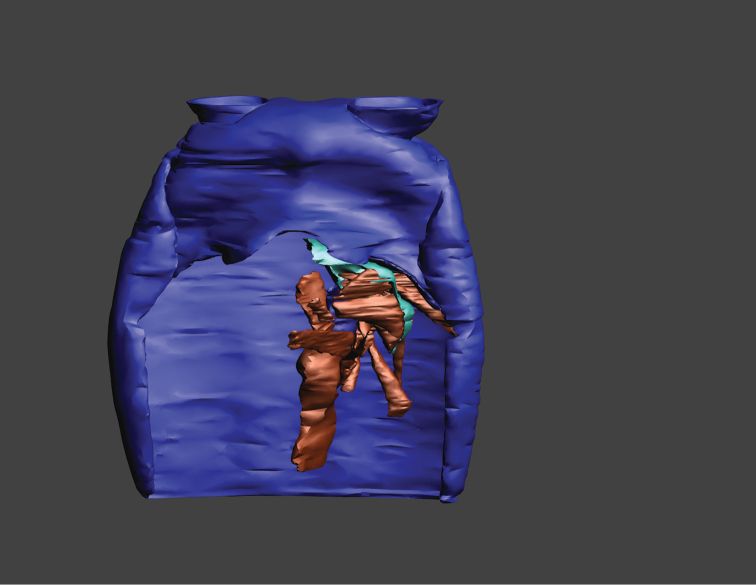
*Hydroschendyla submarina*, surface model of the left mandibulo-tentorial complex in situ. Click on the image to activate the interactive 3D-mode. (download 3D model)

## Results

**Figure 4.**
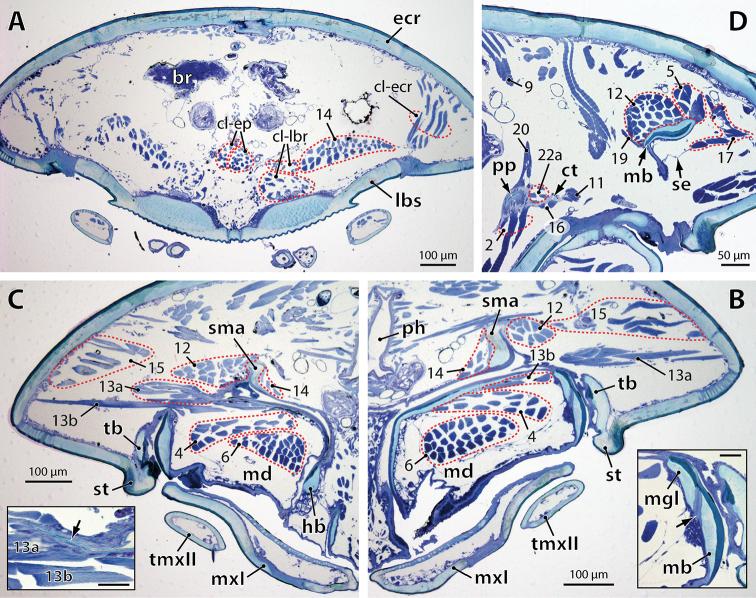
*Dicellophilus carniolensis*, selection of micrographs of transverse sections through the head from anterior to posterior. **A** Section through anterior head part in front of the mandibulo-tentorial complex, highlighting muscles arising from the clypeus **B** Section through the mandibulo-tentorial complex (left side of head) slightly anterior to C; in the inset (scale: 25 µm) the area of flexibility (arrow) between mandibular gnathal lobe and base is magnified. **C** Section through the mandibulo-tentorial complex (right side of head) at the level of the cuticular tendon (arrow in inset; scale: 25 µm) of the mandibular gnathal lobe. **D** Section through posterior part of tentorium and mandible (left side of head), showing collagenous tendon system and mandibular septum. Numbers refer to muscles as listed in Table 1. Abbreviations: **br** brain **cl-ecr** clypeo-epricranial muscle **cl-ep** clypeo-epipharyngeal muscle **cl-lbr** clypeo-labral muscle **ct** collagenous tendon, **ecr** epicranium **hb** hypopharyngeal bar of tentorium **lbs** labral sidepiece **mb** mandibular base **md** mandible **mgl** mandibular gnathal lobe, **mxI** first maxilla **ph** pharynx **pp** posterior process of tentorium, **se** septum of mandibular gnathal pouch **sma** supramandibular arch of tentorium **st** stilus **tb** transverse bar of tentorium **tmxII** telopodite of second maxilla.

In both *Dicellophilus carniolensis* and *Hydroschendyla submarina* the tentorium forms a clasp-like structure dorsally around the mandible (Figs 1–3: *tt*). Its external sclerotisation comprises a short, slender epipharyngeal bar to which the mandibular gnathal lobe is attached via a condyle on its dorsal surface (arrow in Figs 2A, 3A); a slender transverse bar that is laterally jointed to the cephalic pleurite; and a supramandibular arch that behind the mouth opening continues into a hypopharyngeal bar (Fig. 4C). The configuration of these exoskeletal components in ventral view is Y-shaped in *Dicellophilus carniolensis* and T-shaped in *Hydroschendyla submarina*, with the epipharyngeal bar being in either a submarginal (*Dicellophilus carniolensis*) or marginal (*Hydroschendyla submarina*) position relative to the labral sidepieces. Endoskeletal processes of the tentorium comprise a massive ridge (including a frontal process fide Koch 2003) formed by the supramandibular arch, and a flat, wing-like apodeme (posterior process fide Koch 2003) arising at the junction with the hypopharyngeal bar and protruding into the head in a curved, almost vertical orientation. A collagenous bridge interconnecting left and right posterior processes is absent in both species.

### Tentorial muscles

**Figure 5.**
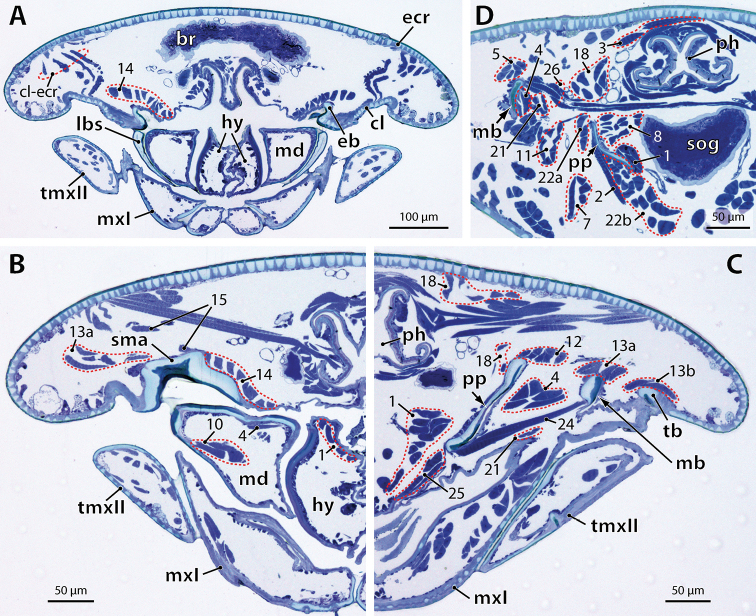
*Hydroschendyla submarina*, selection of micrographs of transverse sections through the head from anterior to posterior. **A** Section through the anterior head part, showing the mandibulo-tentorial complex at the level of the epipharyngeal bar **B** Section through the mandibulo-tentorial complex (right side of head) at the level of the junction between epipharyngeal bar and supramandibular arch. **C** Section through the mandibulo-tentorial complex (left side of head) between the levels shown in B and D. **D** Section through posterior part of tentorium and mandible (right side of head) at the level of the mesial interconnection of the mandibles by muscle 26. Numbers refer to muscles as listed in Table 1. Abbreviations: **br** brain **cl** clypeus, **cl-ecr** clypeo-epicranial muscle **eb** epipharyngeal bar of tentorium, **ecr** epicranium **hy** hypopharynx **lbs** labral sidepiece **mb** mandibular base **md** mandible **mxI** first maxilla **ph** pharynx **pp** posterior process of tentorium **sma** supramandibular arch of tentorium **sog** subesophageal ganglion **tb** transverse bar of tentorium **tmxII** telopodite of second maxilla.

The muscular equipment of the tentorium largely corresponds in the two species studied (Figs 1–3). A total of 17 (*Dicellophilus carniolensis*) or 14 (*Hydroschendyla submarina*) muscles attach at the tentorium, including extrinsic muscles of the mandibles and of the first and second maxillae, two hypopharyngeal muscles, a dilator of the pharynx, muscles arising from the clypeus and the epicranium, and ventral longitudinal muscles (see Table 1). The muscles attach either at the supramandibular arch or at the posterior process; no muscles insert at or originate from the epipharyngeal bar, the transverse bar, or the hypopharyngeal bar. In *Dicellophilus carniolensis* the respective muscles of the first and second maxillae (muscles 2, 7, 11, 16, and 23) partly or entirely arise from collagenous tendons attached to the posterior tip of the tentorial posterior process (Fig. 4D). The homologous muscles in *Hydroschendyla submarina* originate directly from the posterior process (Fig. 5D); this species entirely lacks collagenous tendons associated with the tentorium. Antennal muscles do not arise from the tentorium in either species, but from the clypeus and epicranial wall.

Muscles that in Lithobiomorpha and Scolopendromorpha are thought to move the tentorium are represented in *Dicellophilus carniolensis* and *Hydroschendyla submarina* by the following set:

M.9 is a fan-shaped vertical muscle that originates medio-dorsally from the epicranial wall (Figs 1–3). Its insertion on the tentorium extends from the posterior dorsal edge of the supramandibular arch to the anterior dorsal edge of the posterior process, gradually expanding backwards onto its mesial surface. Relative to the posterior process the origin of this muscle in *Hydroschendyla submarina* lies more posteriorly than in *Dicellophilus carniolensis*.

M.14 is a broad, fan-shaped muscle passing from the clypeus towards the supramandibular arch of the tentorium to insert on its frontal process and mesial surface along its entire length (Figs 1, 2B, 3B, 4A–C, 5A, B).

M.15 passes almost transversely from the lateral epicranial wall towards the supramandibular arch, on which it inserts along its dorsal rim on its lateral face. In *Hydroschendyla submarina* (Figs 3, 5B) the insertion is direct at the tentorium, whereas it is mediated by a collagenous tendon in *Dicellophilus carniolensis* (Figs 2, 4C). In both species the origin of this muscle lies immediately above mandibular muscle 13.

M.18 arises medio-dorsally from the epicranial wall and inserts anteriorly on the mesial surface of the posterior process, in front of the insertion of pharyngeal muscle 8 and below the insertion of tentorial muscle 9 (Figs 1A, 2). The relative positions of these three muscles (8, 9, and 18) are the same in the two species studied, although in *Hydroschendyla submarina* tentorial muscle 18 is far larger than in *Dicellophilus carniolensis* (Fig. 5C, D).

M.22 is a ventral longitudinal muscle attached to the posterior tip of the tentorial posterior process. In both species the muscle consists of two strands. In *Dicellophilus carniolensis* (Figs 1A, 2B, 4D) they insert adjacent to each other at collagenous tendons connected to the posterior process. In *Hydroschendyla submarina* (Figs 1B, 3) the two strands insert directly on the tentorium: a smaller strand (22a) on the dorso-lateral edge of the posterior process above the origin of maxillary muscle 2, and a larger strand (22b) on the ventro-lateral edge of the posterior process below the origin of maxillary muscle 2 (Fig. 5D).

In *Dicellophilus carniolensis* an additional fan-shaped muscle (M.20) passes from the back of the posterior process anteriorly towards the dorsal epicranium (Figs 1A, 2B, 4D). Its attachment at the epicranium lies immediately adjacent (lateral) to the origin of tentorial muscle 9.

### Mandibular muscles

The mandibles of *Dicellophilus carniolensis* and *Hydroschendyla submarina* each consist of a rod-shaped base, somewhat twisted at half length, and a gnathal lobe (for SEM-illustration of the latter see Koch and Edgecombe 2012, their figs 7C and 17C). Both species lack a suture delimiting the two components, but in *Dicellophilus carniolensis* a narrow, circumferential stripe of flexibility within the endocuticle (i.e., an area showing the same histological staining properties as the articular membranes between sclerites) indicates their borders (Fig. 4B, inset). The mandibular musculature differs among the two species in some details.

**Extrinsic mandibular muscles**:

M.3 basically corresponds in the two species in its large extent and transverse orientation. It originates dorso-medially from the epicranial wall and inserts on the dorsal margin of the mandibular base at about half its length (Figs 1, 2A, 3A, 5D).

M.5 is a longitudinal muscle originating dorsally from the posterior wall of the epicranium (Fig. 1). In *Dicellophilus carniolensis* it consists of about 13 smaller bundles; most of them insert one by one on the posterior dorsal margin of the mandibular base, except for three bundles that pass into the concavity of the mandibular base to insert on its internal wall, one far anteriorly to the other two (Fig. 4D). In *Hydroschendyla submarina* this muscle comprises only 6 bundles (Fig. 5D), all of which insert within the mandibular concavity on its internal wall.

M.6 arises from the tentorium and passes towards the gnathal lobe. In *Dicellophilus carniolensis* this muscle is larger than in *Hydroschendyla submarina* and shows a more longitudinal course; it originates from the posterior part of the posterior process, where it covers its entire lateral surface, and inserts on the frontal wall of the gnathal lobe (Fig. 4B,C). In *Hydroschendyla submarina* the origin of this muscle lies anteriorly on the posterior process close to the junction of the hypopharyngeal bar; its insertion extends from the latero-ventral, proximal part of the gnathal lobe onto the anteriormost part of the mandibular base. The course of this muscle accordingly is almost transverse in *Hydroschendyla submarina*.

M.12 passes from the posterior dorsal margin of the mandibular base towards the supramandibular arch of the tentorium. In *Dicellophilus carniolensis* this muscle is larger than in *Hydroschendyla submarina*, its insertion covering the entire lateral surface of the supramandibular arch from the frontal process towards the origin of the posterior process (Figs 1A, 2B, 4B–D). In *Hydroschendyla submarina* the insertion of this muscle on the dorsal rim of the supramandibular arch is restricted to its posterior part while it expands onto the anteriormost dorsal rim of the posterior process (Figs 1B, 3B, 5C).

M.13 is a transverse muscle passing from the cranial wall towards the gnathal lobe. In both species it consists of two sections, a larger fan-shaped dorsal section (13a) and a few bundles below it (13b). In *Dicellophilus carniolensis* (Fig. 1A, 2A) both sections arise dorso-laterally from the epicranial wall; the bundles of the dorsal section (13a) insert on a short, inconspicuous cuticular tendon (Fig. 4C, inset) formed by the dorsal margin of the gnathal lobe, whereas all bundles of the lower section (13b) pass into the concavity of the gnathal lobe to insert directly on its dorsal wall (Fig. 4B,C). In *Hydroschendyla submarina* (Fig. 1B, 3A), the cuticular tendon of the gnathal lobe is larger and more elongate, and all muscle bundles of both sections insert on it. In this species, however, only the bundles of the dorsal section (13a) arise from the latero-dorsal wall of the epicranium, whereas the bundles of the lower section (13b) originate from the ventro-lateral part of the head capsule (Fig. 5B,C).

M.17 interconnects the posteriormost end of the mandibular base with the dorso-lateral wall of the epicranium along a (collagenous?) septum connected to the posterior cuticular, non-sclerotized wall of the mandibular gnathal pouch (Figs 1A, 4D). Although this septum is present in both species, the muscular bundles connected to it in *Dicellophilus carniolensis* are absent in *Hydroschendyla submarina*.

M.19 is an almost vertical muscle in *Dicellophilus carniolensis* (Figs 1A, 4D). It arises from the dorsal epicranial wall and inserts on the ventral margin of the mandibular base at its posterior half. A corresponding muscle seems to be absent in *Hydroschendyla submarina* (but see discussion of its potential homology to mandibular muscle 21)

M.21 is a slender longitudinal muscle composed of four or five small bundles in *Hydroschendyla submarina* (Fig. 5C). It extends from the posterior dorsal wall of the epicranium between the posterior process of the tentorium and the mandibular base towards the gnathal lobe where it inserts proximally at its soft ventral (posterior) wall. A corresponding muscle is absent in *Dicellophilus carniolensis* (but see discussion of its potential homology to mandibular muscle 19).

M.24 interconnects the ventral margin of the mandibular base with the ventral margin of the posterior process. The course of this muscle is almost transverse in *Hydroschendyla submarina* (Figs 3B, 5C) but more oblique in *Dicellophilus carniolensis*. In the latter its insertion at the mandibular base lies more anteriorly than its origin on the posterior process, and it does not attach at the sclerotic part of the mandibular base but on its ventral arthrodial membrane.

M.26 is a transverse muscle in *Hydroschendyla submarina* that interconnects the posterior ends of the left and right mandibular bases (Fig. 5D). The strands of the left and right muscle overlap mesially between the pharynx and the subesophageal ganglion, where they seem to be directly interconnected without any contribution by collagenous tendon. *Dicellophilus carniolensis* lacks this muscle entirely and shares with *Hydroschendyla submarina* the absence of a transverse mandibular tendon.

### Intrinsic mandibular muscles:

M.4 is a large muscle in both species, passing from the posterior end of the mandibular base to the dorsal (anterior) wall of the gnathal lobe (Fig. 3B). In *Dicellophilus carniolensis* this muscle comprises about 15 smaller bundles, all of which extend below mandibular muscle 13 into the gnathal lobe to insert on its proximal, dorsal wall (Fig. 4B,C). In *Hydroschendyla submarina* this muscle instead consists of five larger bundles, three of which extend below the cuticular tendon of mandibular muscle 13 into the gnathal lobe while the other two insert on the base of this tendon (Fig. 5B-D).

M.10 is an intrinsic muscle of the gnathal lobe. In *Hydroschendyla submarina* it connects the lateral distal wall of the gnathal lobe with its proximal, soft mesial wall, immediately in front of mandibular muscle 6 (Fig. 5B). The gnathal lobe of *Dicellophilus carniolensis* lacks any intrinsic muscles.

## Discussion

Verhoeff (1918) was the first to put forth the view that the tentorium of lithobiomorphs and scolopendromorphs is mobile and actively involved in movements of the mandibles. He inferred tentorial mobility in *Lithobius* and *Scolopendra* from dissected heads after removal of all mouthparts behind the mandibles, from which he could ‘observe’ a rotation of the tentorium along the articulation of its transverse bar with the cephalic pleurite by manually pulling on the loosened mandibles and the tentorial posterior process. For geophilomorphs he concluded that movements of the tentorium are primarily maintained, as exemplified for *Himantarium gabrielis* (Adesmata, Himantariidae), albeit in an altered manner and no longer directly accompanying movements of the mandibles due to transformations of the tentorium and mandible morphology correlated with suctorial feeding habits. Attems (1926, 1929) largely adopted Verhoeff’s interpretations.

Manton (1965) basically corroborated Verhoeff’s conclusions with comparative studies on the skeleto-muscular system of the head in centipedes, but she denied any tentorial mobility for geophilomorphs. While including direct observations of mandible movements in live *Lithobius* and *Scolopendra* specimens, she could not directly observe movements of the tentorium but, like Verhoeff, inferred them from the presence and positions of muscles attached at the tentorium, and from manipulation of its skeleton (Manton 1964: 87). Her main reasons for considering the tentorium of geophilomorphs as immobile were based on the view that their heads are primarily adapted to burrowing life habits. Anatomical transformations considered by her as to correlate with burrowing life habits include (i) alteration of the tentorial skeleton, especially absence of a transverse bar; (ii) absence of a “coclypeus” (i.e., paralabial sclerites), “*whose movements are not needed*” (p. 340); (iii) absence of all tentorial muscles (T2-T10 fide Manton) effecting the swing of the tentorium in non-geophilomorph representatives of the Pleurostigmophora; and (iv) simplification of the mandibular musculature, including absence of a transverse mandibular tendon, under restriction of mandible movements to those affected by the mandibular muscles alone. In the light of the current results all these assumptions must be reconsidered.

### Derivation of the tentorial skeleton

The entire construction of the tentorium surprisingly shows no basic difference between *Dicellophilus carniolensis* and *Hydroschendyla submarina*. They indeed seem to differ only in the spatial extension of the tentorium in correspondence to the different shape of the head, in being relatively broad and short in *Dicellophilus carniolensis* versus narrower and more expanded in the longitudinal plane in *Hydroschendyla submarina*. Previous descriptions of the posterior process (“anterior tentorial apodeme” fide Manton 1964, 1965) as rod-shaped proved to be misleading. In both Placodesmata and Adesmata it primarily forms a curved, wing-like apodeme in a similar manner as in non-geophilomorph centipedes. The condition found in *Dicellophilus carniolensis* seems to be plesiomorphic relative to that in *Hydroschendyla submarina* in that remnants of the collagenous, formerly transverse tendon system attached to the posterior processes are preserved in the former.

### Placodesmata

As inferred from *Dicellophilus carniolensis*, the original composition of the tentorium and ancestral arrangement of its cuticular components in Pleurostigmophora are maintained in Placodesmata. A main deviation in Placodesmata concerns the sclerotization of the exoskeletal components that are reduced to slender strips (Koch and Edgecombe 2012, Bonato et al. 2014), as well as a shortening of the epipharyngeal bar. The latter seems to be characteristic for all geophilomorphs. The manner in which the sclerotized lateral end of the transverse bar in placodesmatans bends anteriorly between the cephalic pleurite and the labral sidepiece is reminiscent particularly of the state in scolopendromorphs, in which the lateral end of the transverse bar similarly bends anteriorly to form a hook-shaped point of articulation with the cephalic pleurite (Desbalmes 1992). While the hook-shaped end of the transverse bar in scolopendromorphs borders anterio-mesially against the paralabial sclerite, the antero-mesial border in Placodesmata seems to be formed by the labral sidepieces; paralabial sclerites are not distinct in Placodesmata (see Koch and Edgecombe 2012, their Fig. 2B). Their apparent absence is traditionally interpreted as a loss correlated with a lateral expansion of the labral sidepieces (e.g. Bonato et al. 2014). The alternative interpretation that the paralabial sclerites may be fused with the labral sidepieces has never been taken into consideration, but may be favoured by the labral muscles in *Dicellophilus carniolensis* not showing any marked lateral expansion but instead maintaining a paramedian origin and insertion (Fig. 4A), as in lithobiomorphs (Applegarth 1952, Rilling 1968) and scolopendromorphs (Manton 1964, Desbalmes 1992). This argument, however, is not necessarily conclusive because the clypeo-tentorial muscle (M.14) prevents a lateral expansion of the labral muscles. Since no muscles seem to attach at the paralabial sclerites in any pleurostigmophoran, neither of the two interpretations can unambiguously be favoured by the muscular equipment of the head. Another potential marker to recognise paralabial sclerites is their lateral articulation with the cephalic pleurite in lithobiomorphs and scolopendromorphs. We would expect this articulation to be preserved in placodesmatans were the paralabial sclerites fused with the labral sidepieces. The absence of an articulation between the lateral margins of the sclerite interpreted as a broadened labral sidepiece and the cephalic pleurites in Placodesmata instead supports the traditional interpretation that paralabial sclerites are entirely reduced in Placodesmata.

### Adesmata

There is general agreement on Verhoeff’s (1918) and Manton’s (1965) view that the T-shaped configuration of the exoskeletal components in Adesmata is due to a lateral shift of the entire tentorial complex. The coincident transformation of the tentorial transverse bar and of the paralabial sclerites has, however, been contentious. Three hypotheses have been discussed thus far:

(i) The original articulation between the tentorium (i.e., its transverse bar) and the cephalic pleurite is maintained in adesmatans but has been transformed into a hinge for the gain of an additional articulation between the tentorium and the labral sidepieces; paralabial sclerites are exceptionally identified in adesmatan species such as *Himantarium gabrielis*, in which they are no longer articulated to the labral sidepieces but are anteriorly displaced into a ‘non-functional’ position between the cephalic pleurite and the clypeus (Verhoeff 1918).

(ii) The lateral shift of the tentorium correlates with a loss of both the tentorial transverse bar and the paralabial sclerites as a result of loss of any tentorial mobility (Manton 1965).

(iii) The transverse bar (and its articulation with the cephalic pleurite) is maintained in adesmatans, but it extends straight in line with the epipharyngeal bar, such that the original bifurcation point between these two bars (and their distinct identities) is no longer recognizable. Unified in this manner into a single oblique bar, it positionally replaces the paralabial sclerites, which are lost (Koch and Edgecombe 2012). Based on light microscopical studies of whole-mounts, Bonato et al. (2014, their Table S2) followed Verhoeff’s assumption in considering paralabial sclerites as “*recognizable in the Adesmata*” (coded as present in all sampled adesmatan species), in contrast to Koch and Edgecombe (2012) who could not confirm presence of distinct paralabial sclerites in the position indicated by Verhoeff (1918) in any of the species sampled by them, including *Himantarium gabrielis* (see Koch and Edgecombe 2012, their Fig. 2G). These contrasting views raise the question whether in whole-mounts paralabial sclerites can reliably be distinguished from parts of the tentorium (L. Bonato, pers. comm.). Since the paralabial sclerites in non-geophilomorphs are also articulated to the cephalic pleurites via a hinge-line (e.g., Manton 1965), the difficulty of unambiguously recognizing paralabial sclerites in adesmatan geophilomorphs may indeed be due to the possibility that

(iv) the paralabial sclerites are fused with the tentorium. Loss of the transverse bar would result in the articulation of the tentorium with the cephalic pleurite via its original articulation with the paralabial sclerites.

The cephalic musculature of *Hydroschendyla submarina* does not unambiguously allow a choice of any of the four hypotheses (i-iv) on the transformation of the tentorium in Adesmata. The original muscular equipment of the transverse bar in Pleurostigmophora is absent in this species, which may support the view that the transverse bar is absent as well. Absence of these muscles, however, remains inconclusive, because they are also absent in *Dicellophilus carniolensis*, in which the transverse bar is unambiguously maintained. The tentorial exoskeleton itself in *Hydroschendyla submarina* still raises doubts that the tentorium is coalesced with the paralabial sclerite. This is because the sclerotized oblique bar passing from the cephalic pleurite towards the labral sidepiece proved to merely represent the sharp lateral margin of the tentorium, its external surface being mostly inclined into the depth of the preoral cavity (see Koch & Edgecombe, their Fig. 1F). If the oblique bar – which we consider to comprise both the former transverse and epipharyngeal bars – were to include the paralabial sclerite, one would expect it to form a broader surface onto the ventral head wall posterior to the clypeal sclerotization (i.e., outside the preoral cavity).

We accordingly propose that geophilomorphs overall lack any marker to unambiguously recognize paralabial sclerites. Our data favour a loss of paralabial sclerites in the geophilomorph stem species as a simpler assumption than assuming different transformations of these sclerites in Placodesmata and Adesmata. This view implies that the transverse bar and its articulation with the cephalic pleurite are (primarily) maintained in Adesmata.

### Derivation of the tentorial muscles

The basic set of muscles that in non-geophilomorph Pleurostigmophora are thought to move the tentorium proved to be present in *Dicellophilus carniolensis* and *Hydroschendyla submarina*. Among them, Manton (1965) only recorded the ventral longitudinal muscles for *Orya barbarica* (M.22 in *Dicellophilus carniolensis* and *Hydroschendyla submarina*), as well as a large unlabeled muscle passing from the clypeus to the tentorium “*as in no other chilopod*” (p. 339). This latter muscle corresponds to tentorial muscle 14 in *Dicellophilus carniolensis* and *Hydroschendyla submarina*. Judging from its origin and insertion this muscle can straightforwardly be homologized with tentorial muscles arising in a corresponding position from the clypeus in lithobiomorphs and scolopendromorphs. These comprise a set of muscles (T2-T6 fide Manton) whose insertions are distributed from the anterior tip of the epipharyngeal bar over the supramandibular arch to the lateral tip of the transverse bar. In geophilomorphs they seem to be either unified into a single muscle, or restricted to those muscles formerly inserting close to and on the supramandibular arch at the bifurcation point of the epipharyngeal and transverse bars (i.e., T4 and/or T5). The latter interpretation may be favoured due to the presence of several muscle bundles passing almost vertically from the clypeus to the dorsal epicranial wall in geophilomorphs (Figs 4A, 5A). For these muscle bundles no homologue seems to exist in non-geophilomorph centipedes; with respect to their origin we suspect that they might be derived from tentorial muscles formerly inserting on the epipharyngeal and/or transverse bars. Either transformation of the original set of clypeo-tentorial muscles implies an alignment and expansion of their insertion onto the supramandibular arch and its frontal process.

The set of tentorial muscles arising from the dorsal and dorso-lateral wall of the epicranium in lithobiomorphs and scolopendromorphs (T7-T10 fide Manton) is basically maintained in both *Dicellophilus carniolensis* and *Hydroschendyla submarina*, except for muscle T8 (M.20 in this study), which seems to be lost in *Hydroschendyla submarina*. In both species tentorial muscle 15 deviates from its homologue (T7) in non-geophilomorphs in the shift of its origin towards the lateral epicranial wall, thus acquiring a transverse orientation. Tentorial muscles 9 and 18 deviate from their homologues in non-geophilomorphs (T9 and T10) in the shift of their origin towards a more posterior position, while keeping their distance relative to each other. Compared to *Dicellophilus carniolensis*, their greater distance in *Hydroschendyla submarina* seems to correlate with the stronger longitudinal expansion of the tentorium in this species. The insertion of tentorial muscle 18 (T9) is more derived than in non-geophilomorphs in its expansion onto the mesial surface of the tentorial posterior process. In non-geophilomorphs the corresponding surface of the posterior process mainly serves for insertion of antennal muscles, the insertion of T9 being restricted to the dorsal edge of the posterior process. Its expansion onto the mesial surface in geophilomorphs correlates with the shift of the origin of the respective antennal muscles from the tentorium onto the clypeus.

The arrangement of the clypeo-tentorial, epicranio-tentorial, and ventral longitudinal muscles relative to each other in both *Dicellophilus carniolensis* and *Hydroschendyla submarina* is comparable to a system of six degrees of freedom of movement – its three axes being indicated each by muscles 14 and 22, the set of muscles 9, 18, and 20, and muscle 15, respectively – and basically corresponds to the relative arrangement of their homologues in lithobiomorphs and scolopendromorphs. In applying Manton’s (1964) and Desbalmes’s (1992) functional interpretations of the homologous muscles in scolopendromorphs to *Dicellophilus carniolensis* and *Hydroschendyla submarina*, one would consider muscles 14 and 22 as antagonists for tilting the tentorium forwards and backwards, respectively, whereas muscle 15 and the set of muscles 9, 18, and 20 would be antagonists that tilt the tentorium around its longitudinal axis.

A mobile tentorium was already inferred for Placodesmata by Koch and Edgecombe (2012), but their assumption of a shift of tentorial movements from a longitudinal to a transverse plane in this taxon can no longer be upheld. This assumption was based on consideration of only a few muscles of the mandibulo-tentorial complex, especially tentorial muscles 14 (Koch and Edgecombe 2012: t1) and 15 (t2). The entire set of tentorial muscles revealed in the present study instead questions basic differences in the mobility of the tentorium in Placodesmata and Scolopendromorpha. Adesmata as well does not seem to differ in the mobility of the tentorium, as *Hydroschendyla submarina* proved to largely correspond to *Dicellophilus carniolensis* in the equipment of muscles that are traditionally considered as to primarily cause tentorial movements in other Pleurostigmophora. Koch and Edgecombe (2012) considered the tentorium of adesmatans as immobile because of its transformation correlated with its shift into a more lateral position, with immediate contact to the labrum. This shift and coincident T-shaped configuration of the exoskeletal components necessarily alter the freedom of movements of the tentorium. If this caused the tentorium of adesmatans to be immobile, one would expect the tentorial muscles to be reduced because they are no longer needed. Their presence, however, allows two alternative conclusions: either the tentorium of adesmatans primarily maintains some restricted mobility along a hinge determined by its oblique bar, as advocated by Verhoeff (1918); or the respective tentorial muscles have been misinterpreted as ‘movers’ of the tentorium – not only in Placodesmata, but in Pleurostigmophora as a whole.

Arguments in support of the former interpretation mainly relate to the strength of the muscles and their apparent arrangement as antagonists for each other. Alternatively, if the tentorial muscles are not used to move the tentorium, their main function may rather be to stabilize the tentorium in suspending it firmly at the cranial wall and to keep it in optimal position for its articulation with the mandibles. This new alternative interpretation gains support from current insights on the tentorium in Adesmata, movements of which have traditionally been denied. A case can further be made to question the view that the tentorium performs swinging movements in Pleurostigmophora as a whole. A considerable bulk of muscles originate from the tentorium: apart from extrinsic mandibular muscles these primarily also include antennal muscles, extrinsic muscles of the first and second maxillae, hypopharyngeal muscles, dilators of the foregut, and suspensory muscles of the collagenous transverse tendon system. These muscles drastically reduce the space available for elaborate movements of the tentorium and, because of their origin from the tentorium, its swinging movements would necessarily have an impact on movements of all these head structures. We further suspect that the original interconnection of the posterior processes of the tentorium by the collagenous transverse tendon system in Pleurostigmophora impedes independent movements of the left and right tentorium, and thus also independent movements of the left and right mandible, if swinging movements of the tentorium are really required for the mandibles. We do not deny that the tentorium is flexible to variable degrees in pleurostigmophorans. This flexibility, however, might not be caused by the need to actively contribute to mandibular movements. To allow the mandibles to act against the tentorium, the latter’s flexibility may rather be attributed to a need to stabilize it.

### Derivation of the mandibular muscles

Our current results for *Dicellophilus carniolensis* and *Hydroschendyla submarina* basically confirm Manton’s (1965) view that the mandibular muscles are simplified in geophilomorphs. Her homologies with mandible muscles in non-geophilomorph centipedes, however, are revised as follows.

Among the 14–16 mandibular muscles described for scolopendromorphs (Manton 1964, for *Cormocephalus nitidus*; Desbalmes 1992, for *Scolopendra cingulata* and *Theatops erythrocephalus*), nine homologues are identified in *Dicellophilus carniolensis*, 10 in *Hydroschendyla submarina*. The most remarkable findings pertain to mandibular muscles 17 and 26.

Mandibular muscle 17 was considered by Manton (1965, her muscle 19) to be absent in geophilomorphs, in correlation with the absence of a suspensory sclerite that in Pleurostigmophora primarily connects the posterior tip of the mandibular base along the mandibular gnathal pouch with the epicranial wall. Our results confirm absence of both a suspensory sclerite and associated muscle only for *Hydroschendyla submarina*. Although the suspensory sclerite is also absent in *Dicellophilus carniolensis*, its mandibular gnathal pouch is connected to a septum along which muscle 17 passes backwards to insert on the posteriormost parts of both gnathal pouch and the mandibular base (Fig. 4D). A corresponding muscle was depicted by Manton (1965) for *Orya barbarica*, but she homologized it with a muscle (25) that in scolopendromorphs differs both in origin and insertion. The respective muscle of scolopendromorphs is represented in a corresponding position in both *Dicellophilus carniolensis* and *Hydroschendyla submarina* by mandibular muscle 5. Our results accordingly favour the view that a homologue to mandibular muscle 5 (25 fide Manton) is absent in *Orya barbarica* but instead showing a homologue to mandibular muscle 17 (19 fide Manton in Scolopendromorpha).

Mandibular muscle 26 is remarkable as it seems to represent a remnant of a muscle (32 fide Manton) that in other Pleurostigmophora primarily fills almost the entire concavity of the mandible and interconnects left and right mandible via the transverse mandibular tendon. Its presence in *Hydroschendyla submarina* (Fig. 5D) – not known for any other geophilomorph thus far – provides the first evidence that this muscle is primarily maintained in Geophilomorpha.

The remaining mandibular muscles vary to a lesser degree among geophilomorphs. The strongest adductor of the mandibular gnathal lobe (M.13 in this study; 20 fide Manton) is very much alike in geophilomorphs. As pointed out by Koch and Edgecombe (2012), it deviates from its homologue in lithobiomorphs and scolopendromorphs in its almost transverse orientation, based on the shift of its origin from the dorsal towards the lateral epicranial wall. Its insertion on a cuticular tendon of the mandibular gnathal lobe is more derived in *Dicellophilus carniolensis* (Fig. 4C) than in adesmatans in the reduced size of this tendon and the coincident shift of the insertion of some bundles directly onto the wall of the gnathal lobe. The gnathal lobe of *Dicellophilus carniolensis* is further derived in entirely lacking intrinsic muscles, whereas at least one of the original set in Pleurostigmophora is maintained in Adesmata (M.10 in *Hydroschendyla submarina*, Fig. 5B; 29 fide Manton in *Orya barbarica* and scolopendromorphs). Its absence in *Dicellophilus carniolensis* may correlate with the enormous enlargement of a muscle (M.6 in this study; 27 fide Manton) that passes from the tentorium into the gnathal lobe. As pointed out by Manton (1965), this muscle is generally enlarged in geophilomorphs, but in adesmatans apparently to a lesser degree than in *Dicellophilus carniolensis*. The same holds true for an intrinsic muscle (M.4 in this study; 31 fide Manton) that passes from the mandibular base into the gnathal lobe (Fig. 4B,C). Our results confirm that this muscle is relatively larger and longer in geophilomorphs than in scolopendromorphs, correlated with the shift of its origin to the posterior tip of the mandibular base. Again *Dicellophilus carniolensis* seems to be more derived than adesmatans in the relatively larger size of this muscle and its strongly broadened insertion on the gnathal lobe.

A muscle of the gnathal lobe arising from the dorsal epicranial wall (M.21 in this study and fide Manton) was considered by Manton (1965) to be also enlarged in geophilomorphs, but in *Hydroschendyla submarina* this muscle is similarly slender as in scolopendromorphs, whereas in *Dicellophilus carniolensis* this muscle may be lost. We suspect though that it rather shifted its insertion onto the mandibular base, because in this species a muscle arises from the epicranial wall (M.19 in this study) that attaches to the ventral margin of the mandibular base, a condition not known from any other pleurostigmophoran. Another muscle that inserts on the ventral margin of the mandibular base commonly arises from the tentorium in pleurostigmophorans and is also maintained in *Dicellophilus carniolensis* and *Hydroschendyla submarina* (M.24 in this study; 30 fide Manton). Manton (1965) regarded this muscle as “*indistinguishable*” from longitudinal muscles in *Orya barbarica* (M.12 in this study; 22 and 23 fide Manton) passing from the tentorium to the posterior end of the mandibular base. This view is rejected because these muscles (M.12 and M.24 in this study) have clearly different origins and insertions in both *Dicellophilus carniolensis* and *Hydroschendyla submarina*, as in scolopendromorphs. This rather supports the view that muscle 24 (30 fide Manton) is entirely reduced in *Orya barbarica*.

The longitudinal muscle (M.12) passing from the tentorium to the posterior end of the mandibular base is remarkably larger in *Dicellophilus carniolensis* and *Orya barbarica* than in *Hydroschendyla submarina*. This may support Manton’s view that originally separate muscles (22, 23, and 33 fide Manton) are primarily unified in geophilomorphs but are partly reduced in *Hydroschendyla submarina*. The single muscle arising posteriorly from the dorsal epicranial wall to insert at the dorsal margin of the mandibular base (M.5 in this study) may likewise comprise two formerly separate muscles (24 and 25 fide Manton). These assumptions on unifications of muscles, however, remain uncertain, since in scolopendromorphs some of them seem to be variably reduced (according to Desbalmes 1992: 24 fide Manton in *Scolopendra* and *Theatops*, 33 fide Manton in *Theatops*) and may be convergently reduced in geophilomorphs. The only additional muscle attached to the dorsal margin of the mandibular base (M.3 in this study) seems to be enlarged in *Dicellophilus carniolensis* and *Hydroschendyla submarina* relative to the state of its homologue (26 fide Manton) in *Orya barbarica* and scolopendromorphs.

Whether these transformations of the mandibular musculature in geophilomorphs alter the mandible mechanism to a degree that movements of the tentorium are dispensable remains unclear. This also considers Manton’s (1965) view that the mandibles of geophilomorphs no longer perform see-saw movements. She inferred this mainly from the absence of the transverse mandibular tendon and of the strong mandibular muscle (32 fide Manton) primarily arising from it in Pleurostigmophora. This muscle was interpreted by Manton (1964: 90) to form the “*fulcrum of the see-saw mandibular movements*” in scolopendromorphs. The detection of remnants of it (M.26) in *Hydroschendyla submarina*, albeit devoid of the transverse mandibular tendon, raises doubts on whether abandonment of mandibular see-saw-movements can be generalized for geophilomorphs. Another argument against this view is the presence of a muscle (M.17 in *Dicellophilus carniolensis*, 19 fide Manton) that Manton considered in scolopendromorphs as to be involved in the “recovery movement” of the mandible (Manton 1964: 91), while being absent in geophilomorphs. The differences in the muscular equipment in geophilomorphs overall seem to mainly correlate with the smaller size of the mandibular base that does not provide enough space for all the muscles present in scolopendromorphs. However, all main “functional groups” fide Manton (1964: 91) nevertheless still seem to be represented in geophilomorphs by at least one muscle, albeit in variably altered manners.

## Summary and Perspective

Previous arguments for considering the tentorium of geophilomorphs to be immobile are revised as follows:

(1) Derivation of the tentorial skeleton. – Absence of the transverse bar of the tentorium – until now presumed to determine the axis of tentorial swing in Lithobiomorpha and Scolopendromorpha – is rejected for both Placodesmata and Adesmata. In the former, remnants of the collagenous tendon system proved to be preserved. The muscular system of the tentorium remains ambiguous with regards to the fate of the paralabial sclerites. They may be fused to either the labral sidepiece (Placodesmata), or to the transverse bar of the tentorium (Adesmata), but unambiguous evidence for their presence is lacking. Accordingly, paralabial sclerites may have been lost across Geophilomorpha as a whole.

(2) Derivation of the tentorial muscles. – Entire absence of the muscles that are presumed to effect swinging movements of the tentorium in Lithobiomorpha and Scolopendromorpha is rejected for Geophilomorpha. The respective set of muscles is simplified (either by unification or loss), but the main functional groups fide Manton (1965) are primarily preserved in both Placodesmata and Adesmata. The tentorium of geophilomorphs still serves as the origin for muscles of the mandibles, first and second maxillae as well as both the hypopharynx and pharynx, but no longer for antennal muscles.

(3) Derivation of the mandibular muscles. – A simplification of the mandibular musculature is basically confirmed for Geophilomorpha, but Manton’s (1965) homologisations are partly refuted. The transformation of the mandibular musculature proved to be variable in Placodesmata and Adesmata but in neither instance can a participation of the tentorium in movements of the mandibles be excluded.

As such, our current insights on the morphology of the mandibulo-tentorial complex in geophilomorphs accordingly cause us to doubt that the mandible mechanism differs among pleurostigmophoran centipedes in terms of whether or not movements of the tentorium are required to abduct the mandibular gnathal lobe. With respect to the apparently immobile tentorium in Scutigeromorpha, the size of the mandible and its armature are inconclusive for this problem. The complexity of the muscles involved renders it difficult to unambiguously reveal their interplay and individual function. We therefore think that kinematic studies of living specimens are required to decisively elucidate the functional role of the tentorium during feeding. For this purpose, current advances in 4D in-vivo microtomography (see, e.g., Santos Rolo et al. 2014) seem to provide the most promising technique.

## Supplementary materials

Supplementary material 1 Digital image stack used for 3D-reconstruction of the mandibulo-tentorial complex of *Dicellophilus carniolensis* Data type: QuickTime mov file Explanation note: Series of transverse histological sections from anterior to posterior, provided as movie. This dataset is made available under the Open Database License (http://opendatacommons.org/licenses/odbl/1.0/). The Open Database License (ODbL) is a license agreement intended to allow users to freely share, modify, and use this Dataset while maintaining this same freedom for others, provided that the original source and author(s) are credited.Markus Koch, Johannes Schulz, Gregory D. Edgecombe

Supplementary material 2 Digital image stack used for 3D-reconstruction of the mandibulo-tentorial complex of *Hydroschendyla submarina* Data type: QuickTime mov file Explanation note: Series of transverse histological sections from anterior to posterior, provided as movie. This dataset is made available under the Open Database License (http://opendatacommons.org/licenses/odbl/1.0/). The Open Database License (ODbL) is a license agreement intended to allow users to freely share, modify, and use this Dataset while maintaining this same freedom for others, provided that the original source and author(s) are credited.Markus Koch, Johannes Schulz, Gregory D. Edgecombe

## References

[B1] Applegarth (1952) The anatomy of the cephalic region of a centipede *Pseudolithobius megaloporus* (Stuxberg) (Chilopoda). Microentomology 17: 127–171.

[B2] Attems CG (1926) Chilopoda. In: Kükenthal W Krumbach T (Eds) Handbuch der Zoologie, Vol. 4 Walter De Gruyter, Berlin, 239–402.

[B3] Attems CG (1929) Myriapoda I. Geophilomorpha. In: Schultze FE Kükenthal W (Eds) Das Tierreich, Vol. 52 Walter De Gruyter, Berlin, 388 pp.

[B4] Bonato L Drago L Murienne J (2014) Phylogeny of Geophilomorpha (Chilopoda) inferred from new morphological and molecular evidence. Cladistics 30: 485–507. doi: 10.1111/cla.12060 10.1111/cla.12060 34794246

[B5] Desbalmes G (1992) Funktionsanatomie des Fressapparates der Chilopoda: Die Kopfregion von *Theatops erythrocephala* (C.L. Koch) sowie deren Kauapparat im funktionellen Vergleich mit *Scolopendra cingulata* und *Scutigera coleoptrata*. PhD thesis, University of Vienna, Vienna, Austria.

[B6] Edgecombe GD (2004) Morphological data, extant Myriapoda, and the myriapod stem-group. Contributions to Zoology 73: 207–252.

[B7] Edgecombe GD (2011) Phylogenetic relationships of Myriapoda. In: Minelli A (Ed) Treatise on Zoology – Anatomy, Taxonomy Biology. The Myriapoda. Volume 1 Brill, Leiden, Boston, 1–20. doi: 10.1163/9789004188266_002

[B8] Friedrich F Beutel RG (2008) Micro-computer tomography and a renaissance of insect morphology. In: Stock R (Ed.) Proceedings of SPIE 7078, Developments in X-Ray Tomography VI: . doi: 10.1117/12.794057

[B9] Koch M (2003) Monophyly of the Myriapoda? Reliability of current arguments. African Invertebrates 44: 137–153.

[B10] Koch M Edgecombe GD (2012) The preoral chamber in geophilomorph centipedes: comparative morphology, phylogeny, and the evolution of centipede feeding structures. Zoological Journal of the Linnean Society 165: 1–62. doi: 10.1111/j.1096-3642.2011.00803.x

[B11] Manton SM (1964) Mandibular mechanisms and the evolution of arthropods. Philosophical Transactions of the Royal Society of London Series B 247: 1–183. doi: 10.1098/rstb.1964.0001

[B12] Manton SM (1965) The evolution of arthopodan locomotory mechanisms, Part 8. Functional requirements and body design in Chilopoda, together with a comparative account of their skeleto-muscular systems and an Appendix on a comparison between burrowing forces of annelids and chilopods and its bearing upon the evolution of the arthropodan haemocoel. Journal of the Linnean Society (Zoology) 46: 252–483.

[B13] Rilling G (1968) *Lithobius forficatus*. Grosses Zoologisches Praktikum, Heft 13b. Gustav Fischer Verlag, Stuttgart, 136 pp.

[B14] Santos Rolo Td Ershov A van de Kamp T Baumbach T (2014) In vivo X-ray cine-tomography for tracking morphological dynamics. Proceedings of the National Academy of Sciences of the United States of America 111(11): 3921–3926. doi: 10.1073/pnas.1308650111 24594600 10.1073/pnas.1308650111 PMC3964127

[B15] Shear WA Edgecombe GD (2010) The geological record and phylogeny of the Myriapoda. Arthropod Structure & Development 39: 174–190. doi: 10.1016/j.asd.2009.11.002 19944188 10.1016/j.asd.2009.11.002

[B16] Verhoeff KW (1918) Vergleichende Morphologie und Phylogenie. In: Bronn HG (Ed) , Klassen und Ordnungen des Tierreichs, Band 5, Abteilung 2, Gliederfüssler: Arthropoda, Klasse Chilopoda. Lieferungen 63-101, 1902–1925. Akademische Verlagsgesellschaft, Leipzig, 395–538.

